# Function and Regulation of ALDH1A1-Positive Nigrostriatal Dopaminergic Neurons in Motor Control and Parkinson’s Disease

**DOI:** 10.3389/fncir.2021.644776

**Published:** 2021-05-17

**Authors:** Kathleen Carmichael, Rebekah C. Evans, Elena Lopez, Lixin Sun, Mantosh Kumar, Jinhui Ding, Zayd M. Khaliq, Huaibin Cai

**Affiliations:** ^1^Transgenic Section, Laboratory of Neurogenetics, National Institute on Aging, National Institutes of Health, Bethesda, MD, United States; ^2^The Graduate Partnership Program of NIH and Brown University, National Institutes of Health, Bethesda, MD, United States; ^3^Department of Neuroscience, Georgetown University Medical Center, Washington, DC, United States; ^4^Cellular Neurophysiology Section, National Institute of Neurological Disorders and Stroke, National Institutes of Health, Bethesda, MD, United States; ^5^Computational Biology Group, Laboratory of Neurogenetics, National Institute on Aging, National Institutes of Health, Bethesda, MD, United States

**Keywords:** ALDH1A1, dopamine, Parkinson’s disease, connectivity, motor learning, *substantia nigra*

## Abstract

Dopamine is an important chemical messenger in the brain, which modulates movement, reward, motivation, and memory. Different populations of neurons can produce and release dopamine in the brain and regulate different behaviors. Here we focus our discussion on a small but distinct group of dopamine-producing neurons, which display the most profound loss in the ventral *substantia nigra pas compacta* of patients with Parkinson’s disease. This group of dopaminergic neurons can be readily identified by a selective expression of aldehyde dehydrogenase 1A1 (ALDH1A1) and accounts for 70% of total nigrostriatal dopaminergic neurons in both human and mouse brains. Recently, we presented the first whole-brain circuit map of these ALDH1A1-positive dopaminergic neurons and reveal an essential physiological function of these neurons in regulating the vigor of movement during the acquisition of motor skills. In this review, we first summarize previous findings of ALDH1A1-positive nigrostriatal dopaminergic neurons and their connectivity and functionality, and then provide perspectives on how the activity of ALDH1A1-positive nigrostriatal dopaminergic neurons is regulated through integrating diverse presynaptic inputs and its implications for potential Parkinson’s disease treatment.

## Introduction

Parkinson’s disease (PD), the most common degenerative movement disorder, particularly affects basal ganglia dopamine transmission (Sulzer and Surmeier, 2013; Vogt Weisenhorn et al., 2016). One of the most prominent pathological hallmarks of the disease is a preferential degeneration of dopaminergic neurons (DANs) located in the ventrolateral tier of *substantia nigra pars compacta* (SNc) (Fearnley and Lees, 1991; Kordower et al., 2013). While a member of aldehyde dehydrogenase family genes termed murine class 1 (cytosolic) aldehyde dehydrogenase (AHD2) or aldehyde dehydrogenase 1A1 (ALDH1A1) was reported some time ago to be selectively expressed by a subpopulation of DANs in the rodent ventral SNc (McCaffery and Drager, 1994), it is until 20 years later that research in post-mortem human brains demonstrates a conserved topological distribution of ALDH1A1-positive DANs in the human SNc as well as a more severe loss of ALDH1A1-positive nigrostriatal DANs (ALDH1A1^+^ nDANs) in PD patients compared to the ALDH1A1-negative ones (Liu et al., 2014). ALDH1A1 is a key enzyme to mediate the biosynthesis of retinoic acids (McCaffery and Drager, 1994) and catabolism of reactive dopamine metabolites (Marchitti et al., 2007; Burke, 2010) in DANs. The reduction of ALDH1A1 expression may contribute to the etiopathogenesis of PD (Galter et al., 2003; Mandel et al., 2007; Werner et al., 2008; Grunblatt et al., 2017), whereas an increase of ALDH1A1 levels protects against dopaminergic neurodegeneration (Cai et al., 2014; Liu et al., 2014). Although the expression and biochemical function of ALDH1A1 protein is extensively documented, less is known regarding the molecular, electrophysiological, anatomical, and physiological properties of ALDH1A1^+^ nDANs. We believe that a further in-depth study of ALDH1A1^+^ nDANs will bridge the gap toward a cell-type specific understanding of neural circuit mechanisms and treatment of PD.

## ALDH1A1 Defines and Protects a Nigrostriatal Dopaminergic Neuron Subpopulation

The nigrostriatal DANs are diverse in nature and can be categorized into groups of distinct subpopulations based on location, gene expression profiles, electrophysiological properties, morphology, projection pattern, physiological functions, and vulnerabilities to various diseases (Liu et al., 2014; Poulin et al., 2014; Lerner et al., 2015; Menegas et al., 2015; Evans et al., 2017; Hook et al., 2018). Traditionally, midbrain DANs can be divided into three main subgroups, retrorubral field (RRF, A8), SNc (A9), and ventral tegmental area (VTA, A10), in human and rodents (Bentivoglio and Morelli, 2005; Vogt Weisenhorn et al., 2016). In the post-mortem brains of PD patients, the most profound loss of DANs has been seen in the ventral tier of SNc (Fearnley and Lees, 1991; Kordower et al., 2013). Further studies have demonstrated that these ventral DANs can be molecularly defined by a selective expression of ALDH1A1 (Cai et al., 2014; Liu et al., 2014). ALDH1A1 belongs to ALDH superfamily genes, which consist of 19 members in human genome (Koppaka et al., 2012) and 20 members in mouse genome (Cai et al., 2014). ALDH1A1 is predominantly and highly expressed by the ventral DANs in human and mouse SNc, suggesting its distinctive role in the function and survival of ventral DANs (Cai et al., 2014; Liu et al., 2014). As a multifunctional enzyme in DANs, ALDH1A1 mediates the synthesis of retinoic acids important for the differentiation of DANs during development (Jacobs et al., 2007). ALDH1A1 is also suggested to conduct the alternative synthesis of inhibitory transmitter GABA in DANs (Kim et al., 2015). More importantly, ALDH1A1 oxidizes the highly reactive dopamine catabolic intermediate dopamine-3,4-dihydroxyphenylacetaldehyde (DOPAL) and protects ALDH1A1^+^ nDANs against DOPAL-induced cytotoxicity (Marchitti et al., 2007; Burke, 2010). A recent study suggests that DOPAL can be actively produced in DANs when the monoamine oxidase (MAO)-mediated dopamine oxidation is employed in ATP production in mitochondria (Graves et al., 2019). DOPAL is highly reactive and a lack of ALDH1A1 may lead to accumulation of DOPAL, which has been shown to promote cytotoxic polymerization of PD-related α-synuclein and compromise the functions of proteins important in the activity and survival of DANs (Rees et al., 2009). Accordingly, ALDH1A1^+^ nDANs are less vulnerable to α-synuclein–mediated neurodegeneration compared with the ALDH1A1-negative ones in α-synuclein transgenic mice, while genetic deletion of *Aldh1a1* exacerbates DAN loss (Liu et al., 2014). Downregulation of *ALDH1A1* mRNA and protein levels along with severe loss of DANs has also been reported in the ventral SNc of post-mortem PD brains (Galter et al., 2003; Mandel et al., 2007; Werner et al., 2008). The reduction of ALDH1A1 expression in PD may weaken the protective function of ALDH1A1 in the ventral tier of SNc and predispose these neurons to degeneration at the later stages of disease (Cai et al., 2014). Therefore, a profound reduction of ALDH1A1 expression may represent the turning point toward pathogenicity of ventral SNc DANs undergoing neurodegeneration in PD and ALDH1A1 expression level and activity may be extrapolated as a useful biomarker to monitor the progression of the disease as well as potential therapeutic targets (Cai et al., 2014).

The ALDH1A1^+^ DANs account for 63% of SNc, 32% of VTA and 5% of RRF DANs in mouse brains (Wu et al., 2019). The ALDH1A1^+^ DANs also make up for 72% of SNc DANs in human brains (Liu et al., 2014), while the percentages in other midbrain brain regions remain to be determined. The ALDH1A1^+^ DANs in VTA regions exhibit distinct connectivity patterns compared to their counterparts in SNc (Wu et al., 2019); however, little is known about their functional contribution to any behavioral phenotypes. Therefore, we focused the present review on the SNc ALDH1A1^+^ DANs only.

## Molecular Characteristics of ALDH1A1-Positive Nigrostriatal Dopaminergic Neurons

**FIGURE 1:**
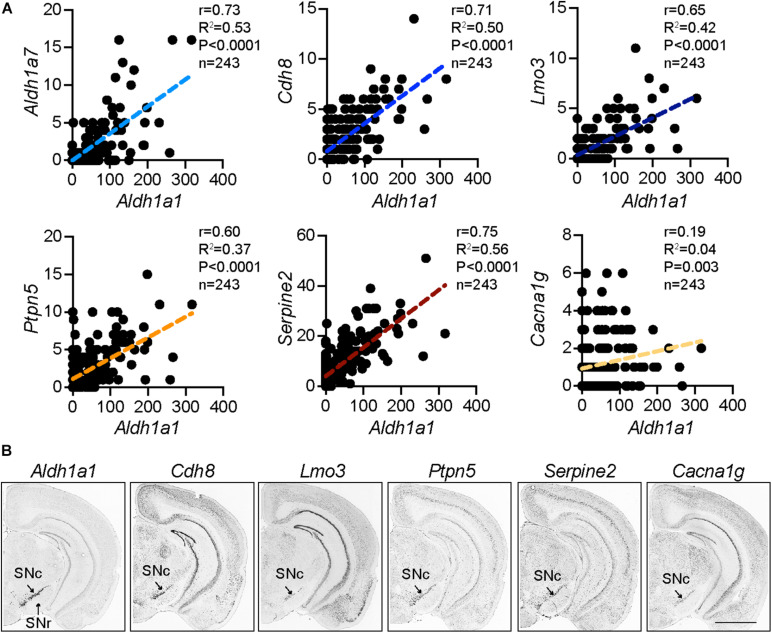
Distinct molecular signature of ALDH1A1^+^ nDANs. **(A)** Correlation of *Aldh1a1* expression with a selective set of genes in mature mouse DANs from a single cell RNA sequencing study (La Manno et al., 2016). **(B)** *In situ* hybridization of *Aldh1a1* and correlated genes in the SNc of adult mouse brains (Allen Brain Atlas).

The ALDH1A1^+^ nDANs are closely clustered in the ventral tier of SNc (Wu et al., 2019). The most distinctive genetic markers for this subtype of DANs in rodents are *Aldh1a1* (McCaffery and Drager, 1994; Liu et al., 2014; Poulin et al., 2014) and *Aldh1a7* (Cai et al., 2014). The *Aldh1a7* gene is located next to the *Aldh1a1* in the mouse chromosome 19 and is highly homologous to the *Aldh1a1*. *Aldh1a7* gene is absent in the human genome, which may contribute to the higher sensitivity of human DANs to dopamine-related cytotoxicity and PD-related genetic insults (Cai et al., 2014; Liu et al., 2014). Recently, single-cell RNA-sequencing (scRNA-seq) in combination with various mRNA fluorescence *in situ* hybridization methods provide unprecedented molecular details for diverse DAN subpopulations at different developmental stages (Poulin et al., 2014; La Manno et al., 2016; Hook et al., 2018; Tasic, 2018; Tiklova et al., 2019). A number of genes are highly correlated with the *Aldh1a1* expression in the rodent DANs (La Manno et al., 2016), including *Lmo3*, *Cdh8*, *Serpine2*, *Ptpn5*, and *Aldh1a7* (Figure 1A), which also display a similarly restricted expression pattern in the ventral SNc as *Aldh1a1* mRNAs in mouse brains (Allen Brain Atlas) (Figure 1B), indicating these genes are among the molecular signature of ALDH1A1^+^ nDANs. By contrast, there is no or extremely low expression of *calbindin* in the ALDH1A1^+^ nDANs (Poulin et al., 2014; La Manno et al., 2016), which may serve as a useful marker for ALDH1A1-negative nigrostriatal DANs. In a recent scRNA-seq study with postnatal day 60 to 70 mouse brain, *Vglut2*, *Cbln4*, *Neurod6*, and *Tacr3* were added as additional markers for the mature ALDH1A1^+^ nDANs (Saunders et al., 2018). Moreover, based on the unique expression of *Vcan*, *Anxa1*, and *Grin2C*, the ALDH1A1^+^ nDANs can be further divided into three sub-populations (Saunders et al., 2018). The gene expression studies lay the foundation for later functional characterization of distinct ALDH1A1^+^ nDANs subpopulations under normal and disease-related conditions.

## Electrophysiological Properties of ALDH1A1-Positive Nigrostriatal Dopaminergic Neurons

**FIGURE 2:**
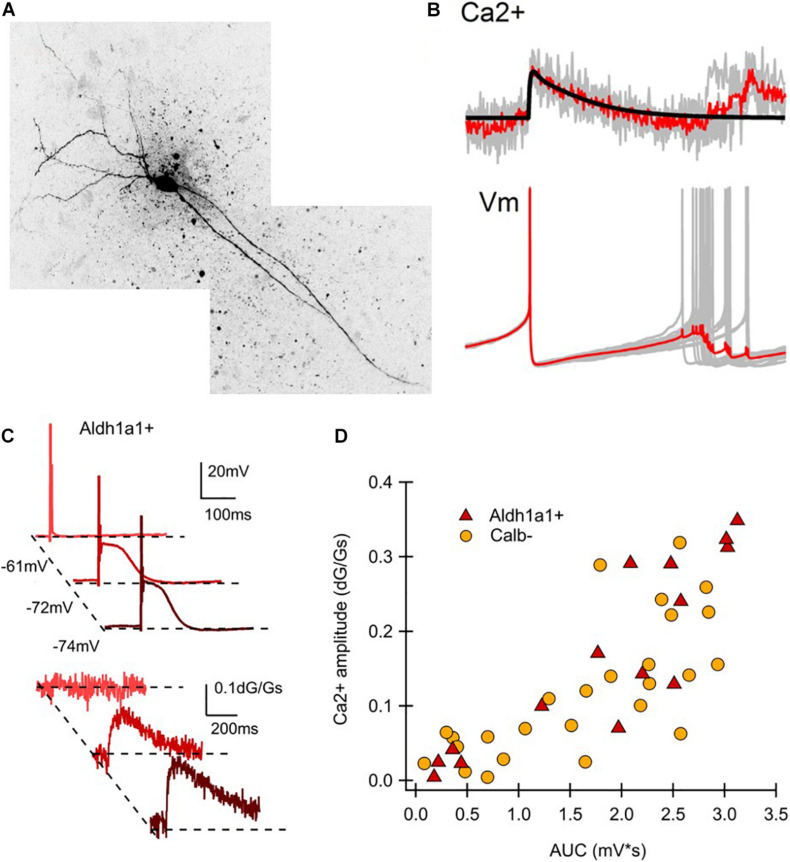
Electrophysiological characteristics of ALDH1A1^+^ nDANs. **(A)** Two-photon image of a ALDH1A1^+^ nDAN. **(B)** Average calcium transient (above) and action potential (below) shape during tonic firing for a ALDH1A1^+^ nDAN. **(C)** Hyperpolarization-dependent after depolarizations (above) and corresponding dendritic calcium transients (below) for a ALDH1A1^+^ nDAN. **(D)** Amplitude of the calcium transient from a potential of −80 mV graphed by the area under the curve (AUC) for the corresponding low-threshold depolarization. Calb^–^, Calbindin-negative population (data re-graphed from Evans et al., 2017, **(D)**, adult heated calbindin-negative population).

**FIGURE 3:**
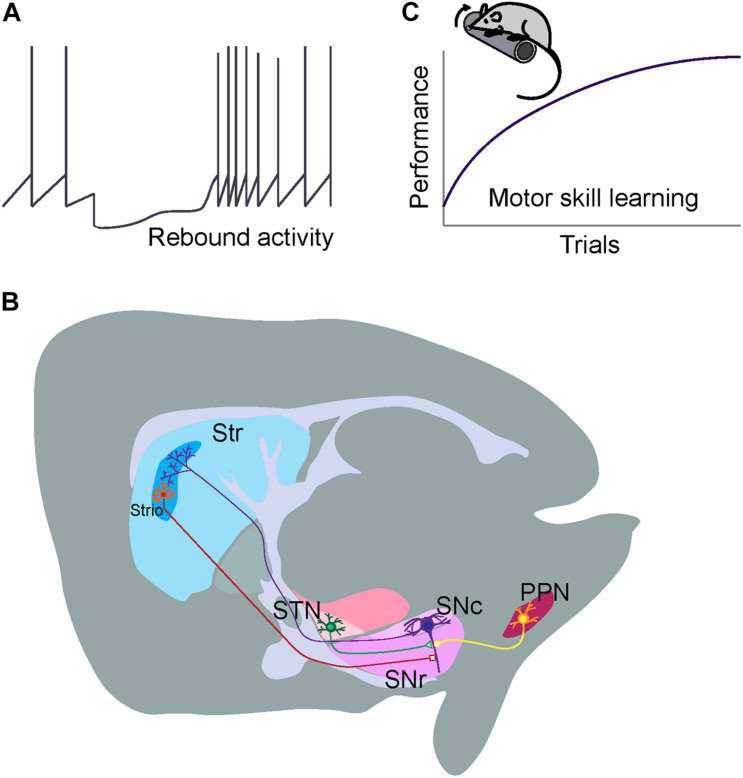
Distinct electrophysiology, connectivity, and functionality of ALDH1A1^+^ nDANs. **(A)** Rebound activity of ALDH1A1^+^ nDANs following dSPN inhibition. **(B)** Major inhibitory and excitatory presynaptic inputs to the ALDH1A1^+^ nDANs. **(C)** A critical involvement of ALDH1A1^+^ nDANs in regulating the vigor of movement during the acquisition of motor skills.

ALDH1A1^+^ nDANs exhibit distinct electrophysiological properties and rebound more readily from hyperpolarization (Evans et al., 2017). To interrogate the electrophysiological properties of ALDH1A1^+^ nDANs, we performed whole-cell recoding of tdTomato-labeled neurons in SNc slices of 2–5-month-old *Aldh1a1*^+/CreERT2^/Ai9 mouse brains (Figure 2A). The ALDH1A1^+^ nDANs fired spontaneous action potentials (APs) at a rate of 0.5–6 Hz (1.8 ± 0.4 Hz, *n* = 15). They had characteristically broad APs (1.9 ± 0.1 ms, *n* = 15) with a height of 68.5 ± 2.5 mV, an input resistance of 327.5 ± 26.2 MΩ, and a capacitance of 63.3 ± 5.23. During each AP, a calcium transient of 0.033 ± 0.005 dG/Gs (*n* = 12) was apparent in the dendrites (Figure 2B). Furthermore, the ALDH1A1^+^ nDANs shared many characteristics with the calbindin-negative neurons, which populate the ventral tier of the SNc (Evans et al., 2017). Specifically, the ALDH1A1^+^ nDANs had a large voltage “sag” during hyperpolarization (17 ± 1.6 mV) indicative of a strong hyperpolarization cation current (*I*_*h*_, Figure 2C). In addition, these neurons demonstrated large low-threshold depolarizations [area under the curve (AUC) from −80 mV membrane potential: 1.8 ± 0.28 mV ^∗^s, *n* = 15], indicative of strong T-type calcium channel activity. Using two-photon calcium imaging (see Evans et al., 2017 for methods), we found that these low-threshold depolarizations were accompanied by large dendritic calcium transients (0.17 ± 0.032 dG/Gs, *n* = 15) (Figure 2C). When graphing the size of the low threshold depolarization (AUC) by the calcium amplitude for each cell, labeled neurons from the *Aldh1a1*^+/CreERT2^ mouse show a strong similarity to unlabeled (calbindin-negative) neurons from the calbindin-Cre mouse (Figure 2D), indicating that these neurons represent overlapping populations. Compared to the calbindin-positive DANs in the dorsal tier of SNc, the calbindin-negative DANs exhibit increased sensitivity to excitatory inputs following dopamine-mediated autoinhibitory stimulation, which then trigger large dendritic calcium transients likely through T-type calcium channels (Evans et al., 2017). The ventral DANs also display distinct rebound activity in response to the inhibitory inputs from striatal projection neurons (SPNs) (Evans et al., 2020; Figure 3A). Therefore, the ALDH1A1^+^ nDANs appear to differ substantially in their responses to both excitatory and inhibitory presynaptic inputs compared to the calbindin-positive DANs, which may contribute to their distinct physiological functions in motor control and learning. This absence of calbindin in the more PD-vulnerable ALDH1A1^+^ nDANs also suggests the relevance of calcium buffering in PD pathophysiology (Surmeier and Schumacker, 2013).

## Projection Pattern of ALDH1A1-Positive Nigrostriatal Dopaminergic Neurons

The ALDH1A1^+^ nDANs exhibit a distinct projection pattern in the rostral and dorsal portions of dorsal striatum (DS), including both the dorsomedial striatum (DMS) and dorsolateral striatum (DLS) (Sgobio et al., 2017; Poulin et al., 2018; Pan et al., 2019; Wu et al., 2019). In parallel with the location of their cell bodies in the SNc, the projection of ALDH1A1-positive axon fibers is arranged along the same medial to lateral axis in the DS (Wu et al., 2019). By contrast, ALDH1A1-positive nigrostriatal DANs in the more caudal SNc regions tend to innervate the more rostral striatal regions (Wu et al., 2019). In DS, the densities of ALDH1A1^+^ nDAN axon fibers display a gradient change along the dorsal to ventral and rostral to caudal axes (Poulin et al., 2018; Wu et al., 2019). Noticeably, the ALDH1A1^+^ nDANs project heavily to the dorsal portion of DS, the striatal region that is also heavily innervated by the sensorimotor cortices (Hintiryan et al., 2016); as well as the rostral striatal regions, which also receive mixed innervations from both associative and sensorimotor cortices (Hintiryan et al., 2016). The convergence of diverse cortical glutamatergic and midbrain dopaminergic inputs in the rostral DS indicates the functional importance of this striatal region in motor control and learning. Additionally, a small fraction of ALDH1A1-positive axon fibers converges to the striosome (or called patch) compartments in DS (Sgobio et al., 2017; Poulin et al., 2018; Wu et al., 2019; Figure 3B). The functional significance of this specific innervation remains to be determined. It needs be pointed out that ALDH1A1-positive DANs are also comprised of heterogenous subtypes and an individual subtype may possess distinct connectivity and functionality. With the increasing availability of single cell RNAseq data, we expect additional genetic markers could be identified to further molecularly define different subpopulations of ALDH1A1-positive DANs for more in-depth circuit studies.

## Monosynaptic Inputs Onto ALDH1A1-Positive Nigrostriatal Dopaminergic Neurons

ALDH1A1-positive DANs receive the majority of monosynaptic inputs from the striatum (Wu et al., 2019). Compared to the non-specified nigrostriatal DAN total populations (Watabe-Uchida et al., 2012), ALDH1A1^+^ nDANs receive more innervations from ventral striatum and hypothalamus, but less from cerebral cortices, pallidum, amygdala, and midbrain regions (Wu et al., 2019). Furthermore, ALDH1A1^+^ nDANs appear to form reciprocal innervation with SPNs in the dorsal regions of DS (Wu et al., 2019). This reciprocal connection between ALDH1A1^+^ nDANs and SPNs may constitute a feedback loop for timely regulating the dopamine release and neuron activity in motor control. Both striosome and matrix SPNs innervate ALDH1A1^+^ nDANs in the ventral SNc (Wu et al., 2019). Some of the striosome SPN axons are intermingled with the dendrites of ventral ALDH1A1^+^ nDANs perpendicularly protruding in the *substantia nigra pars reticulata* (SNr) and form this so-called striosome-dendron bouquet structure (Crittenden et al., 2016; Evans et al., 2020; Figure 3B), which may establish a unique striatonigral circuit for unspecified physiological functions. Besides the inhibitory presynaptic inputs from SPNs, ALDH1A1^+^ nDANs receive the majority of excitatory monosynaptic inputs from subthalamus nucleus (Wu et al., 2019). The impact of both inhibitory and excitatory presynaptic inputs on the function and regulation of ALDH1A1^+^ nDANs will be discussed in the later sections.

## Dopamine Release Dynamics of ALDH1A1-Positive Nigrostriatal Dopaminergic Neurons

There has been no direct quantification of dopamine release from the axon terminals of ALDH1A1^+^ nDAN in DS. Since ALDH1A1-positive dopaminergic axons converge onto striosome compartments in the DLS (Sgobio et al., 2017), afferent stimulus-evoked dopamine release was compared between striosome and surrounding matrix compartments by fast scan cyclic voltammetry in a line of striosome reporter mice (Salinas et al., 2016; Sgobio et al., 2017), in which the green fluorescent protein-marked striosomes can be readily identified under epifluorescence microscope (Davis and Puhl, 2011; Sgobio et al., 2017). The amplitude of evoked dopamine release is lower in striosome compared to matrix compartments (Salinas et al., 2016; Sgobio et al., 2017). Genetic deletion of *Aldh1a1* selectively enhances dopamine release in striosomes, suggesting that ALDH1A1 actively regulates dopamine release in ALDH1A1–positive fibers projecting to the DLS striosomes, but not the surrounding matrix area (Sgobio et al., 2017). In addition, pharmacological inhibition of dopamine reuptake also leads to more dopamine release in the striosomes than in the proximal matrix areas (Davis and Puhl, 2011; Sgobio et al., 2017), correlated with a higher dopamine transporter (DAT) level in the ALDH1A1-positive axon terminals in the striosomes (Sgobio et al., 2017). DAT mediates the uptake of 1-methyl-4-phenyl-1,2,3,6-tetrahydropyridine (MPTP)-derived neurotoxin cation 1-methyl-4-phenylpyridinium (MPP^+^) in DANs (Frim et al., 1994; Langston, 2017). The higher content of DAT in ALDH1A1^+^ nDANs might be attributable to the increased sensitivity of ALDH1A1^+^ nDANs to MPTP-mediated cytotoxicity (Poulin et al., 2014). By contrast, neither dopamine D2 autoreceptors nor nicotinic acetylcholine receptors appear to differentially regulate dopamine release in striosome and matrix compartments (Sgobio et al., 2017). The differential expression of ALDH1A1 and other proteins for dopamine synthesis, packaging, reuptake, and degradation in ALDH1A1^+^ nDANs may contribute to the distinct dopamine release dynamics (Sgobio et al., 2017). With the availability of Cre mouse lines that specifically target gene expression in the ALDH1A1^+^ DANs (Poulin et al., 2018; Wu et al., 2019) and genetically encoded dopamine sensors (Patriarchi et al., 2018; Sun et al., 2018), a direct measurement of dopamine release from ALDH1A1^+^ nDANs in live behaving mice may provide new insight into how the dynamic of dopamine release contributes to the physiological function of ALDH1A1^+^ nDANs.

## Physiological Function of ALDH1A1-Positive Nigrostriatal Dopaminergic Neurons

It has been generally accepted that the nigrostriatal DAN-mediated dopamine transmission is essential in regulating the vigor of movement (Mazzoni et al., 2007; Dudman and Krakauer, 2016). Movement vigor represents a key element of movement manifested with speed, amplitude, or frequency; while motor motivation drives movement vigor (Mazzoni et al., 2007; Dudman and Krakauer, 2016). The nigrostriatal DAN-mediated dopamine transmission is proposed to signal the motor motivation (Dudman and Krakauer, 2016), which provides the theoretical framework to explain why the degeneration of nigrostriatal DANs in PD patients leads to reduced movement vigor (Mazzoni et al., 2007; Dudman and Krakauer, 2016). A causal relationship has been established in rodents between the activity of nigrostriatal DANs before movement initiation and the probability and vigor of future movements (da Silva et al., 2018). However, a selective ablation of ALDH1A1^+^ nDANs in mouse brains only moderately reduces the occurrence of high-speed walking when the mice are free to choose movement speed in Open-field test (Wu et al., 2019). Compared to a modest reduction in high-speed walking, the ALDH1A1^+^ nDAN-ablated mice display much more severe impairments in accelerating rotarod test, in which the mice have to move at an instructed and gradually increased speed. These observations suggest that ALDH1A1^+^ nDANs play a more critical role in supporting goal-oriented actions that demand strong motor motivation.

ALDH1A1^+^ nDANs are also implicated in motor skill learning (Wu et al., 2019). Motor skill is regarded as the ability to select and execute goal-directed actions and act over a range of vigor (Dudman and Krakauer, 2016). Motor skill learning, a product of both learning actions and the capacity to flexibly parameterize their execution, is required for optimizing movements in every aspect of life (Kantak and Winstein, 2012; Dudman and Krakauer, 2016). The associate cortex-DMS and sensorimotor cortex-DLS circuits function coordinately during the acquisition of skilled movements (Corbit et al., 2017; Kupferschmidt et al., 2017), in which dopamine dynamically modulates synaptic strength of cortical and striatal neurons and serves as a reinforcement learning signal in the DS (Valentin et al., 2016). The repeated rotarod test is a well-adopted motor training paradigm to examine the motor skill learning in rodents (Sommer et al., 2014), which includes both the initial acquisition phase to optimize the foot placement on the rotating rod and the later retention phase to maintain the optimal stepping practice (Cao et al., 2015). The ablation of ALDH1A1^+^ nDAN in mouse brains completely abolish the improvement of motor performance in the rotarod motor skill learning tests (Wu et al., 2019). Further study demonstrates that ALDH1A1^+^ nDAN are essential in the acquisition of skilled movements, but not for the maintenance of acquired motor skills (Wu et al., 2019). These observations support the notion that nigrostriatal dopamine released from ALDH1A1^+^ nDANs functions as a key feedback signal for the cortico-striatal network-mediated reinforcement learning (Valentin et al., 2016). Together, we hypothesize that ALDH1A1^+^ nDAN-mediated dopamine transmission provides the implicit motor motivation and means to gain new motor skills through improvement of movement vigor during the learning phase (Figure 3C).

Systemic administration of levodopa or dopamine receptor agonists allows the ALDH1A1^+^ nDAN-ablated mice to walk faster but fail to improve the motor skill learning (Wu et al., 2019). Similarly, dopamine replacement therapy is also less effective in treating the PD patients with learning and memory deficiency (Emre, 2003; Heremans et al., 2016). These findings suggest that dynamic dopamine release from ALDH1A1^+^ nDANs is a key requirement for the learning process (Helie et al., 2015). ALDH1A1^+^ nDANs may integrate diverse presynaptic inputs from basal ganglion and other brain regions to dynamically regulate the neuronal activity and dopamine release during the learning process.

## Functional Regulation of ALDH1A1-Positive Nigrostriatal Dopaminergic Neurons

ALDH1A1^+^ nDANs receive monosynaptic inhibitory GABAergic inputs from DS, external globus pallidus (GPe) and other brain regions (Wu et al., 2019). Both striosome and matrix direct pathway SPNs (dSPNs) innervate ALDH1A1^+^ nDANs (Wu et al., 2019). However, striosome dSPNs may supply a higher ratio of direct inputs on nigrostriatal DANs compared to matrix dSPNs (McGregor et al., 2019). Striosome dSPNs can induce a pause-rebound firing pattern exclusively in ventral nigrostriatal DANs through GABA-B receptors on dendron bouquets as a potential mechanism to control plasticity of dopamine secretion (Evans et al., 2020). The GPe, however, does not exhibit a similar firing pattern when stimulating GABA-A receptors on ventral nigrostriatal DANs (Evans et al., 2020), suggesting a differential functional output of GABA signaling in subpopulations of nigrostriatal DANs depending on the origin of the signal. Since the ventral nigrostriatal DANs may not necessarily be all ALDH1A1-positive, future studies will be needed to further elucidate the synaptic transmission of ALDH1A1^+^ nDANs by taking advantage of recently developed *Aldh1a1*-Cre knock-in mouse lines (Poulin et al., 2018; Wu et al., 2019). Selective ablation of dSPNs in mice also completely prevents the improvement in performing rotarod motor skill learning task (Durieux et al., 2012), suggesting that the dSPN-ALDH1A1^+^ nDAN circuit is essential for motor skill learning (Figure 3B). Partial ablation of μ-opioid receptor (MOR1)-positive striosome SPNs with the toxin dermorphin-saporin seems to mainly affect the motor improvement in the later training sections (Lawhorn et al., 2009). The role of striosome dSPNs in motor skill learning, however, remains to be determined.

The subthalamic nucleus (STN), cortex, and pedunculopontine nucleus (PPN) all provide excitatory inputs to the ALDH1A1^+^ nDANs, but the major source of excitatory input to ALDH1A1^+^ nDANs comes from neurons projecting from the STN (Wu et al., 2019; Figure 3B). While the role of glutamatergic input to ALDH1A1^+^ nDANs in regulating dopamine signaling and ALDH1A1^+^ nDAN activity has not been well-characterized, the nature of glutamatergic input in the central nervous system as a whole and its role in synaptic plasticity suggests it is important for learning and adapting behavior. Treatment for PD patients that involves deep brain stimulation of the STN suggests that STN input in particular plays an important role in regulating at least some of the behaviors that are disrupted in PD (Dayal et al., 2017), emphasizing the importance of understanding the role of glutamatergic input. Similar to the lack of work investigating the effect of glutamatergic regulation on ALDH1A1^+^ nDAN activity and signaling, there is also insufficient work isolating the behavioral effects of pharmacologically or genetically altering glutamatergic input to ALDH1A1 + nDANs. For example, although behavioral work with mice suggests that impaired glutamatergic input to midbrain DANs disrupts performance in tasks related to effort and incentive but not motor coordination or reward learning (Hutchison et al., 2018), the behavioral consequences of glutamatergic input onto ALDH1A1^+^ nDAN in particular is not clear. This inability to discriminate the effects of different types of DANs is extremely prevalent in studies investigating the role of glutamatergic input onto midbrain DANs. Although many experiments leave us unable to decipher the role of glutamatergic input to ALDH1A1^+^ nDANs in isolation, the findings from such experiments can still give us insight into how glutamatergic input to midbrain DANs in general is important. Hopefully in the future we can use that knowledge to see how ALDH1A1^+^ nDAN activity and their glutamatergic regulation work in support or in opposition to other neurons with respect to motor skill learning and other PD-related behaviors.

## Conclusion and Future Perspectives

Previous studies demonstrate that ALDH1A1^+^ nDANs are preferentially degenerated in PD, the most common degenerative movement disorder (Cai et al., 2014; Liu et al., 2014). Further studies in rodent models reveal distinct molecular composition, electrophysiological properties, connectivity and functionality of this DAN subpopulation (Poulin et al., 2014, 2018; La Manno et al., 2016; Evans et al., 2017, 2020; Sgobio et al., 2017; Pan et al., 2019; Wu et al., 2019). There is still much to learn about the physiological function and regulation of ALDH1A1^+^ nDANs and how to compensate for the lost function of those neurons as occurred in PD. While the inputs to ALDH1A1^+^ nDANs have been well-characterized (Watabe-Uchida et al., 2012; Wu et al., 2019), how the relevant inputs from each of the identified brain areas regulate the activity and physiological function of ALDH1A1^+^ nDANs has not been completely elucidated. Parsing out these specific anatomical sources of presynaptic inputs and their relative functional contributions in regulating ALDH1A1^+^ nDANs will allow us to better understand the ALDH1A1^+^ nDAN-mediated circuit mechanism of motor control.

The importance of regulated dopamine release by nigrostriatal DANs, particularly ALDH1A1^+^ nDANs, may explain why so many therapies for PD that largely focus on simply supplementing lost dopamine fail to fully restore behavioral deficits in patients, including learning and memory deficits (Emre, 2003; Rochester et al., 2010; Beeler et al., 2012). While dopamine levels alone may help with alleviating or reversing some symptoms, evidence now seems to suggest that more complex or demanding tasks such as motor learning not only require dopamine release but need tightly regulated dopamine release as learning occurs. A better understanding of how ALDH1A1^+^ nDANs integrate diverse presynaptic inputs to regulate dopamine release may also provide insight into which behavioral tests are most effective at studying the more nuanced and complex symptoms of nigrostriatal dopamine loss and seeing which treatment interventions most fully restore those symptoms in PD.

The revelation of preferential vulnerability of ALDH1A1^+^ nDANs in PD promotes ongoing efforts in understanding cell-type and neural circuit specific mechanism of the disease. By taking advantage of newly developed single cell RNA sequencing, CRISPR/Cas9 gene editing, optogenetics, chemogenetics, and live imaging with genetically encoded indicators techniques, we expect that increasing knowledge will be gained on how different subtypes of DANs contribute to different aspects of behavioral phenotypes. A further emphasis on system and behavioral neuroscience may provide new mechanistic insights into designing novel therapeutic strategies for PD treatment.

## Author Contributions

HC outlined the article, wrote the introduction, physiology, function, and perspectives sections, as well as prepared the figures. KC wrote the main regulation and conclusion sections. EL contributed to the regulation section. MK contribute to introduction section. LS wrote the molecule section. JD contributed to the gene expression analyses. RCE and ZMK contributed to the electrophysiology analyses. All authors contributed to the article and approved the submitted version.

## Conflict of Interest

The authors declare that the research was conducted in the absence of any commercial or financial relationships that could be construed as a potential conflict of interest.
